# Comparison of two diagnostic protocols in the management of possible cardiac chest pain: One follow-up study in Iran

**DOI:** 10.22088/cjim.12.2.148

**Published:** 2021-03

**Authors:** Kamran Heidari, Mahbube Asghari Arani, Mehdi Sheibani, John W Pickering, Arezoo Chouhdari

**Affiliations:** 1 Skull Base Research Center, Loghman Hakim Medical Center, Shahid Beheshti University of Medical Sciences, Tehran, Iran; 2 Department of Internal Medicine, Shahid Beheshti University of Medical Sciences, Tehran, Iran; 3 Cardiovascular Research Center, Shahid Beheshti University of Medical Sciences,Tehran, Iran; 4 Clinical Research Development Center of Loghman Hakim Hospital, Shahid Beheshti University of Medical Sciences, Tehran, Iran; 5 Emergency Department, Christchurch Hospital and Department of Medicine, University of Otago Christchurch, New Zealand; 6 Department of Health and Community Medicine, School of Medicine, Shahid Beheshti University of Medical Sciences, Tehran, Iran

**Keywords:** Protocols, Management, Chest pain, Acute coronary syndrome

## Abstract

**Background:**

Chest pain indicating acute coronary syndrome (ACS) accounts for approximately 5-10% of presents in the emergency departments (EDs). Rapid decision making is very important because longer hospital stay is associated with higher financial burden. The aim of this study was to compare current practice with a 2-hour accelerated diagnostic protocol (ADP) to manage chest pain in patients suspected to have ACS.

**Methods:**

This is a longitudinal follow-up study on 900 patients with negative troponin measured on entrance to the ED and initially low-risk for myocardial infarction according to the emergency department of chest pain assessment score (EDACS) at the Loghman Hakim Hospital, Tehran, Iran in 2018. Patients were divided in two groups (based on odd or even days at admission time) at a ratio of 2:1 (i) current protocol with a second troponin measuring after 6 hours and (ii) ADP with a second troponin measured after 2 hours. Major adverse cardiac events (MACE) associated factors assessed in two groups over 30-days.

**Results:**

Totally, the rate of return to EDs with the major adverse cardiac events was 4% (n=24) in the current protocol group and 1% (n=1) in the ADP group within 30 days. The odds ratio for MACE in 30 days in the current protocol was 4.3 times more than ADP group (95% CI: 1.28-14.56, OR: 4.33, p:0.02). In multivariable logistic regression analysis, this estimation for the current protocol was 4.10 times more than comparison group (95% CI: 1.23-13.81, OR: 4.10, p:0.01).

**Conclusion:**

A 2-hour ADP in patients at low-risk for myocardial infarction by EDACS had fewer adverse follow-up events than the current protocol.

Acute coronary syndrome (ACS) is a term used to define a range of conditions associated with sudden reduced blood flow to the heart. ACS usually starts suddenly and symptoms include angina, tightness or burning, pain radiating from the chest to the shoulders, dyspnea or unexplained fatigue (1, 2). ACS accounts for approximately 5% to 10% of annual referrals to emergency departments (EDs) and up to 25% of hospital admissions (3). The importance of emergency care of patients suspected to have an ACS is because ACS may lead to a cardiac arrest or even death. Therefore, a sufficient diagnostic strategy is one which ensures safe disposition of these patients (4). Accepted guidelines for management of ACS include laboratory evaluation of cardiac troponin (cTn) soon after the onset of symptoms. Troponin (cardiac I and T) is a specific indicator of damage to the myocardium.

Elevations of cTn in blood is used for differential diagnosis of unstable angina from myocardial infarction in people with chest pain or ACS (5, 6). Diagnosis of patients at high risk of ACS is well known; however, the majority of chest pain presentations are low-risk patients and diagnosis of these cases remains a challenge. The current protocol for management of ACS patients in emergency cases, is serial measurements of non–high sensitivity cTn during 6 to 12 hours from the time the symptom starts or presentation to the ED. This prolonged procedure leads to a traffic jam of patients in ED that is accompanied with unnecessary costs for both the health system and the patient, and finally risk of increased mortality (7, 8). Therefore, an improved protocol to more rapidly screen patients referred to ED because of chest pain who have low risk of cardiac events would appear to have advantages if it is shown to be both safe and effective. Recently, a new strategy, called an accelerated diagnostic protocol (ADPs), has been trialed and shown to safely lead to earlier discharge of low risk patients. This new method, according to the scoring system, monitors patients at low risk for coronary events within 2 hours. Two ECGs and two troponin and CPK-MB tests check within 2 hours. Therefore, the use of ADPs results in potentially shortening of hospital length of stay and helps better management and follow up of patients (9). It seems that a safe and efficient program is required to manage potential ACS patients referring to EDs. 

The aim of the present study was to compare the use of an ADP with conventional diagnostic assessment in an Iranian emergency department setting. We assessed comparative safety by following low-risk patients with the recurrence of possible major adverse cardiac events for 30 days. 

## Methods

This study was a single-center longitudinal following designed with convenience sampling method to compare two follow up pathways which include cardiac Troponin measurement in the current (0, 6 hours) vs. new (0, 2 hours) protocol for the diagnostic assessment of patients with positive cardiac chest pain. All patients with chest pain who had a negative troponin (cTn <0.01 µg/l; VIDAS Troponin l Ultra, bioMérieux, France) test and normal echocardiography (ECG) result on arrival to the emergency department were eligible. The inclusion criteria were age over 18 years, chest pain, and neck, jaw or arm discomfort with unknown source. Exclusion criteria were ST segment elevation myocardial infarction (STEMI) present on any electrocardiograph (ECG), patients with proven or suspected non-coronary pathology as the cause of chest pain, patients who will require admission regardless of a negative cTn due to other medical conditions or need for other investigations, patient (or legal representative) unable or unwilling to provide informed consent. A total number of 967 patients with chest pain had no ECG change, so normal cTn test was presented in the emergency department of Loghman Hakim Hospital, Tehran, Iran in 2018. They were divided into two groups according to the odd and even days at admission time. Current and accelerated diagnostic (ADP) protocols with approximate ratio (640 patients in current and 327 in new protocol) to follow- up cTn test and ECG changes at two times of 2 and 6 hours after admission. Overall, 67 patients were excluded due to abnormal cTn or ECG changes at the 2 and 6 hours. The remaining patients (600 in current and 300 in new or ADP protocols) were followed up for 30 days recording return visits with symptoms of ACS. All 900 patients were examined by a physician and a clinical history was taken which included recording of risk factors for ACS: hypercholesterolemia, high blood pressure, diabetes, smoking, family history of cardiovascular disease, and history of heart attack. A clinical history was recorded which included the type of pain including whether it radiated to the shoulder and arm, was reproduced on palpation, was reproduced on inspiration, was associated with abnormal sweating (diaphoresis). An Emergency Department Assessment of Chest Pain Score (EDACS) was calculated for each patient (10). The ADP criteria for patient classification as low risk and calculation of EDACS has been described previously (9, 10). If EDACS was less than 16 and the ECG and cTn concentrations were normal, then the patient was considered to be low risk and eligible for discharge. Online calculators for EDACS are available at http://edaculator.adelaideemergencyphysicians.com and https://www.mdcalc.com/emergency-department-assessment-chest-pain-score-edacs. Major adverse cardiac events (MACE) within 30 days assessed and include death (unless non-cardiac), cardiac arrest, emergency revascularization procedure, cardiogenic shock, ventricular arrhythmia needing intervention, high degree atrioventricular block needing intervention and myocardial infarction(10). All patients completed informed consent form at the beginning of the study. This study was approved by the Ethics Committee of Shahid Beheshti University of Medical Sciences, Tehran, Iran (Ethical Code: IR.Sbmu.MSP.REC.1397.460). 

**Statistical analysis**. For the report of descriptive results, the mean, standard deviation and number and percentage were used. For data analysis, chi square and Fisher’s Exact also independent t-test and Mann-Whitney U test were used. To predict possible factors, related to the returning of patients within 30 days, multivariable logistic regression analysis was performed (odds ratio, 95% confidence interval). As well as the hazard function curve for comparison of the effect of 2 diagnostic protocol on “return in 30 days” and flow chart of this study indicated. Total analysis executed by SPSS software Version19. 

## Results

There were 967 eligible patients of whom 67 were excluded resulting in 300 (33.3%) patients in the ADP group and 600 (66.7%) in the current protocol group. In this study, 303(50.5%) and 145(48.31%) of patients in current and ADP groups, were males. Chest pain was mostly seen in the 18-45-year group. Past medical history and the clinical manifestation are reported in table1. Demographic and clinical history variables were compared with the patient's return with cardiac symptoms and signs within 30 days in both groups and the results are presented in table 2.

**Table 1:** Past medical history and clinical manifestation in two groups of patients: Current and ADP groups

**Parameter**	**Current protocol group** **(N=600, 66.7%)**	**ADP group** **(N=300, 33.3%)**
Hypercholesterolemia Yes No	110(18.3) 490(81.7)	50(16.7) 250(83.3)
Hypertension Yes No	294(49) 306(51)	147(49) 153(51)
Diabetes Yes No	165(27.7) 437(72.5)	72(24) 228(76)
Smoking Yes No	208(34.7) 392(65.3)	100(33.3) 200(66.7)
Positive Family History Yes No	198(33) 402(67)	107(35.7) 193(64.3)
Past MI Yes No	34(5.7) 566(94.3)	10(3.3) 290(96.7)
Past Revascularization Yes No	19(3.2) 581(96.8)	13(4.3) 287(95.7)
Pain radiated to arm or shoulder Yes No	381(63.5) 218(36.5)	196 (65.3) 104 (34.7)
Pain produced with palpation Yes No	120(20.0) 480(80.0)	67(22.3) 233(77.7)
Pain worsened by inspiration Yes No	154(25.7) 446(74.3)	105(35) 195(65)
Diaphoresis Yes No	131(21.8) 469(78.2)	66(22) 234(78)

**Table 2:** Association between MACE in 30 days and clinical findings

**Variable**	**MACE within 30 days** **Current protocol** **N(%)**		**P value**	**MACE within 30 days** **ADP** **N(%)**		**P value**
	**Yes** **24(4)**	**No** **576(96)**		**Yes** **3(1)**	**No** **297(99)**	
Sex Male Female	16(66.7) 8(33.3)	287(49.8) 289(50.2)	0.1	2(66.7) 1(33.3)	143(48.1) 154(51.9)	0.5
Age groups 18-45 Over 45	6(25) 18(75)	247(42.9) 327(57.5)	0.04	0(0) 3(100)	159(53.5) 138(46.5)	0.1
Hypercholesterolemia Yes No	2(8.3) 22(91.7)	108(18.8) 468(81.3)	0.2	0(0) 3(100)	50(16.8) 247(83.2)	0.4
Hypertension Yes No	11(45.8) 13(54.2)	283(49.1) 293(50.9)	0.8	2(66.7) 1(33.3)	145(48.8) 152(51.2)	0.6
Diabetes Yes No	2(20.8) 19(79.2)	159(27.6) 417(72.4)	0.6	1(33.3) 2(66.7)	71(23.9) 226(76.1)	0.4
Smoking Yes No	7(29.2) 17(70.8)	199(34.5) 377(65.5)	0.5	1(33.3) 2(66.7)	99(33.3) 198(66.7)	0.9
Positive Family History Yes No	6(25) 18(75)	192(33.3) 384(66.7)	0.3	2(66.7) 1(33.3)	105(35.4) 192(64.6)	0.2
Past MI Yes No	1(4.2) 23(95.8)	33(5.7) 543(94.3)	0.7	0(0) 3(100)	16(3.4) 287(96.6)	0.7
Past Revascularization Yes No	2(8.3) 22(91.7)	17(3) 559(97)	0.2	0(0) 3(100)	13(4.4) 284(95.6)	0.1
Pain Radiated to arm or shoulder Yes No	17(70.8) 7(29.2)	364(63.3) 211(36.7)	0.5	2(66.7) 1(33.3)	194(65.3) 103(34.7)	0.9
Pain Produced with palpitation Yes No	6(25) 18(75)	114(19.8) 462(80.2)	0.6	1(33.3) 2(66.7)	66(22.2) 231(77.8)	0.5
Pain worsened by breathing Yes No	5(20.8) 19(79.2)	149(25.9) 427(74.1)	0.5	1(33.3) 2(66.7)	104(35) 193(65)	0.9
Diaphoresis Yes No	5(20.8) 19(4.1)	126(21.9) 450(78.1)	0.8	2(66.7) 1(33.3)	64(21.5) 233(78.5)	0.1
EKG change Yes No	6(25) 18(75)	75(13) 501(87)	0.09	2(66.7) 1(33.3)	44(14.8) 253(85.2)	0.1
Total EDCAS Score Mean ± SD	8.93±4.74	8.03±5.33	0.5	10±5.56	7.27±5.67	0.6

**Figure 1:**
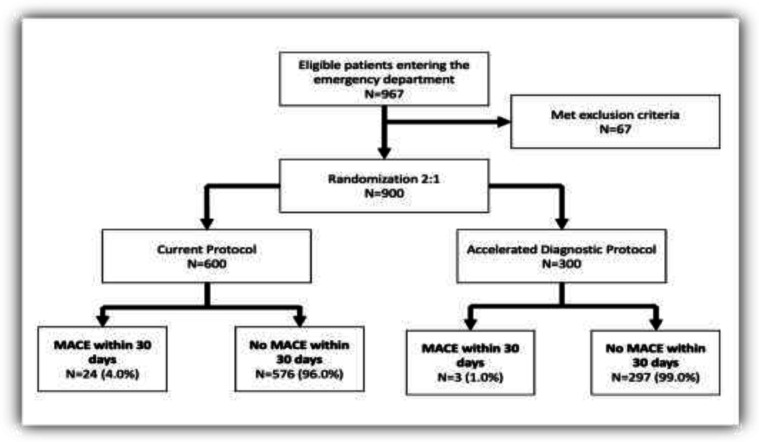
Flow chart of study and results

**Figure 2:**
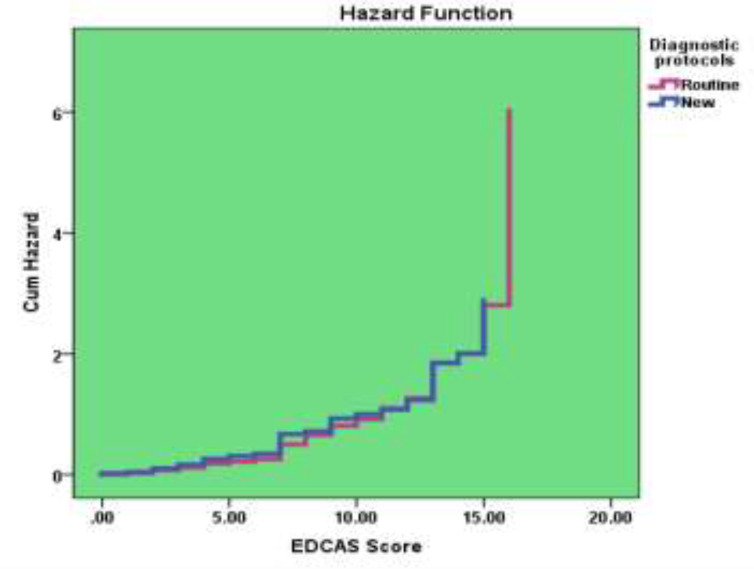
The hazard function for comparison of the effect of 2 diagnostic protocol on " Return in 30 days "

Within each pathway there was no apparent difference between those with and without a Major Adverse Cardiac Event for any variable with the possible exception of age groups (table 2). Results of full and final model of multivariable logistic regression analysis to predict the return of symptoms within 30 days are shown in tables 3 and 4. Flow chart of this study and hazard function curve for the comparison of the effect of 2 diagnostic protocol on “return in 30 days” plotted (figures 1 &2). The rate of MACE within 30 days was 4 times less for the EDACS pathway (1%) than the current pathway (4%). With increasing EDCAS score, the chance to return to the hospital within 30 days in both groups (0/2 hours and 0/6 hours) increased.

**Table 3:** Full model of multivariable logistic regression to evaluate association between return to hospital within 30 days and variables studied

**Variables**	**OR (95% CI)**	**P value**
Revascularization Yes No(reference)	3.11(1.27-7.59)	0.01
Age group 18-45 46-65 ≥66(reference)	2(0.7-5.7) 2.8(0.9-8.07)	0.1 0.5
Protocol Routine (0.6 hours) New (0.2 hours)(reference)	4.33(1.28-14.56)	0.02

**Table 4:** Final model of multivariable logistic regression to predict return to hospital within 30 days

**Variables**	**OR (95% CI)**	**P value**
Revascularization Yes No(reference)	3.28(1.35-7.96)	0.02
Protocol Routine (0.6 hours) New (0.2 hours)(reference)	4.10(1.23-13.81)	0.01

## Discussion

In this longitudinal follow-up study, patients with a new accelerated diagnostic protocol serial troponin measurement on arrival in the ED and 2 hours later discharge earlier with normal ECG and cTn fewer returned to the hospital with cardiac events during 30 days follow-up via phone. This statistical result was interesting to us. There is currently no universally accepted definition for ‘low-risk’ patient for ACS, but there are many options available. Some of these are high-sensitivity troponin assays. Troponin assays are still the most common tests for decision making of patients admitted with acute chest pain. 

When serial troponin sampling combine with ECG test, the detection sensitivity goes up and false negative decrease. Recently, a new method of patients with acute chest pain follow-up (0 and 2 hours CTn assays) has integrated into clinical practice in the whole of New Zealand (EDACS-ADP or ADAPT-ADP), Queensland, Australia (ADAPT-ADP), and three hospitals in North Carolina, USA (HEART-ADP). Amongst 19,803 patients assessed with EDACS-ADP or ADAPT-ADP and discharged as low-risk, there were no 30-d MACE events where the protocol had been correctly followed (9-10). In a study conducted by Than et al., the benefit of rapid ADP for chest pain in low risk patients for early discharge was surveyed. As a result, out of 1975 patients, 302 (15.3%) had MACE. 392 patients (20%) were classified as ADP-based low-risk group. One patient (0.25%) had MACE, 99.7% ADP sensitivity, 99.7% negative predictive value, 23.4% specificity and 19.05 positive predictive value (9). 

Another study evaluated advanced and validated EDACS and the 2 hour rapid diagnostic protocol. 1974 and 608 trials approved, EDACS-ADP 42.2% (99.0% sensitivity, 49.9% specificity) and 51.3% (100.0% sensitivity, 59.0% specificity) classified as low-risk MACE, intraclass correlation coefficient for patients classified as a low risk it was 0.87. As a result, EDACS-ADP identified approximately half of patients admitted to emergency room with chest pain as having low risk of short-term high-sensitivity MACE, a similar improvement similar to that observed previously (4). No study has demonstrated outside of the European, North American, or Australasian contexts, the safe utilization of these pathways. 

To the best of our knowledge, this present study is the first to apply an ADP utilizing EDACS outside of these jurisdictions. Our hypothesis was that the 2-hour ADP would perform as well as the 6-hour current protocol. Perhaps, surprisingly given that all patients in both arms of the study were initially (after one troponin) regarded as possible low-risk using EDACS, the 2-hour ADP resulted in fewer patients with a follow-up MACE. 

It is most important that we can be confident, the ADP is at least as safe in patients with angina who did not feel chest pain at time of referral, ECG is usually normal unless there are other cardiovascular problems in the past. During periods of pain, falling or increase in the ST segment may be observed. To diagnose these changes, an ECG test may be requested. If the marked ECG changes are documented (usually more than 1 mm below the baseline or ST fall), angiography is considered for diagnosis. Even continuous monitoring of blood pressure and pulse can lead to conclusions about angina (11-12). In the present study, ECG changes were used as an important diagnostic tool in patients. The results of our study indicate that 86.6% and 84.7% of patients with normal ECG were in the current protocol and the 2-hour protocol, respectively. 

In patients with ECG changes, the most frequent changes were T invert and T tall changes in the current protocol and 2-hour protocol, respectively. In most patients, pain radiation in the shoulder and arm was seen as the main symptom of the disease. In the case of pain associated with shortness of breath and breathing exacerbation, the majority of patients in both groups were negative and around 78% of the patients did not show abnormal sweating. The most important of limitation in this study was following up via phone. Therefore, recall bias may happen. 

In conclusion overall, the results of this study showed that the recurrence in patients in a 30-day period was more common in current protocol than a 2-hour ADP protocol. This suggests that it would be safe to adopt the accelerated diagnostic protocol with EDACS in patients presenting with chest pain to Iranian emergency departments. This would reassure many patients much earlier that they are not having a myocardial infarction and would free up physicians and nurses to spend more time treating higher-risk patients.
